# Drop-on-demand cell bioprinting via Laser Induced Side Transfer (LIST)

**DOI:** 10.1038/s41598-020-66565-x

**Published:** 2020-06-16

**Authors:** Hamid Ebrahimi Orimi, Sayadeh Sara Hosseini Kolkooh, Erika Hooker, Sivakumar Narayanswamy, Bruno Larrivée, Christos Boutopoulos

**Affiliations:** 1 Centre de Recherche Hôpital Maisonneuve-Rosemont, Montréal, Canada; 2 Department of Mechanical, Industrial and Aerospace Engineering, Concordia University, Montréal, Canada; 3 Department of Ophthalmology, Faculty of Medicine, University of Montreal, Montréal, Canada; 4 Departent of Mechanical Engineering, SRM University, AP Amaravati, India; 5 Department of Molecular Biology, University of Montreal, Montreal, Quebec Canada; 6 Institute of Biomedical Engineering, University of Montreal, Montreal, Quebec Canada

**Keywords:** Biomedical engineering, Physics, Tissue engineering

## Abstract

We introduced and validated a drop-on-demand method to print cells. The method uses low energy nanosecond laser (wavelength: 532 nm) pulses to generate a transient microbubble at the distal end of a glass microcapillary supplied with bio-ink. Microbubble expansion results in the ejection of a cell-containing micro-jet perpendicular to the irradiation axis, a method we coined Laser Induced Side Transfer (LIST). We show that the size of the deposited bio-ink droplets can be adjusted between 165 and 325 µm by varying the laser energy. We studied the corresponding jet ejection dynamics and determined optimal conditions for satellite droplet-free bioprinting. We demonstrated droplet bio-printing up to a 30 Hz repetition rate, corresponding to the maximum repetition rate of the used laser. Jet ejection dynamics indicate that LIST can potentially reach 2.5 kHz. Finally, we show that LIST-printed human umbilical vein endothelial cells (HUVECs) present negligible loss of viability and maintain their abilities to migrate, proliferate and form intercellular junctions. Sample preparation is uncomplicated in LIST, while with further development bio-ink multiplexing can be attained. LIST could be widely adapted for applications requiring multiscale bioprinting capabilities, such as the development of 3D drug screening models and artificial tissues.

## Introduction

Cell bioprinting technologies aim to build living constructs with long term mechanical and biological stability suitable for transplantation, as well as to provide improved 3-dimensional (3D) drug discovery models^1,2^. Central goal in cell-bioprinting is the positioning of multiple cell types on a supporting substrate in a precise manner. Post printing cell viability and spatial resolution are key determinants for the overall efficacy of the printing process. The main bioprinting technologies include drop-on-demand approaches^3^, such as ink-jet printing^4,5^ and laser-induced forward transfer (LIFT)^6^, as well as microextrusion^2,7^. Depending on the printing mechanism, these technologies present partial only compatibility with available bio-ink formulations, with the bio-ink viscosity being the limiting factor^2^. For example, ink-jet printing is limited to the 3.5–12 mPa s viscosity range and microextursion from 30 mPa s to >6 × 10^7^ mPa s.

LIFT does not use a nozzle. Such an implementation enables printability for an extended bio-ink viscosity range (1–300 mPa/s). In LIFT, a focused laser beam is used to propel a small quantity of a bio-ink film, previously spread on a transparent donor substrate, towards a receiving substrate. It has been successfully employed for 2D printing of a wide range of biomaterials, including living cells^8^, proteins^9^, isolated photosynthetic materials^10^ and nucleic acids^11^, with marginal cell viability compromise^12–19^. Despite these significant developments, LIFT has yet to broadly reach tissue engineering laboratories. The main limitation of this technology is the necessity to apply and maintain a thin and uniform bio-ink film (5–20 µm) on the donor substrate. This step is technically challenging and limits 3D printing capabilities of LIFT. Indicatively, 3D bio-printing of a 1 cm^3^ construct would require the preparation of a 1 m^2^ bio-ink film.

Laser-induced flow focusing has been used to print droplets of model (viscosity: 2–210 mPa s) and protein solutions^20^. This approach has been initially implemented for the generation of supersonic microjets aiming to needle-free drug injection^21–24^. Laser-induced flow focusing uses laser-induced bubble generation close to a liquid/air interface (i.e., the distal end of liquid filled microtube) to produce a micro-jet via the displacement of a concave shaped liquid surface. This technology has been largely exploited for supersonic jet generation, but it has not been tested for cell bioprinting.

**Figure 1:**
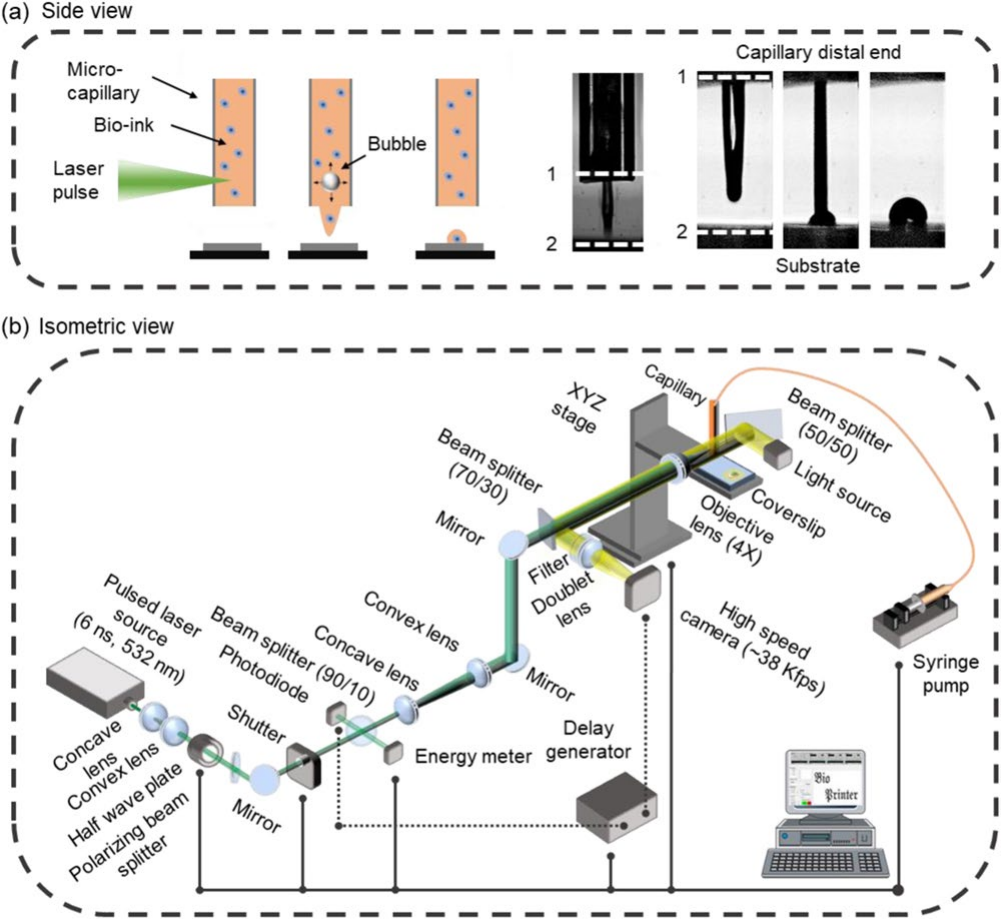
Overview of LIST (**a**) Schematic side view representation of LIST bioprinting (left) and indicative high-speed imaging of bio-ink ejection (right). The distal end of the capillary and the substrate have been identified with numbers. (**b**) Detailed schematic of the LIST bioprinting setup.

In this work, we present a non flow focusing variation of this approach as a method to print cells. The method, coined Laser Induced Side Transfer (LIST), uses low energy laser pulses to generate a transient microbubble at the distal end of a glass microcapillary supplied with bio-ink. This causes the ejection of cell-containing micro-jet perpendicular to the irradiation axis (Fig. 1a). We studied the jet ejection dynamics in LIST and determined optimal conditions for uniform bioprinting of human umbilical vein endothelial cell (HUVEC) containing drops. We demonstrated droplet bio-printing up to a 30 Hz repetition rate and showed that LIST-printed HUVECs presented marginal loss of viability and maintained their abilities to migrate, proliferate and form intercellular junctions.

## Results and Discussion

### Laser induced side transfer (LIST)

In LIST, cell-containing droplets are generated by focusing a 6 ns laser pulse (wavelength 532 nm, pulse duration 6 ns, energy per pulse 50 to 150 μJ) in a hollow square glass capillary filled with bio-ink. The irradiance at the focal plane is tuned to exceed the cavitation threshold of the bio-ink, resulting in the generation of transient spherical bubble (Fig. 1a). Bubble expansion propels the bio-ink toward the capillary opening, resulting in the ejection of cell-containing microjet (Fig. 1a). The LIST setup consists of three main parts: a) a laser beam delivery system, b) a drop-on-demand unit, and c) a micro-jet visualization system (Fig. 1b). The setup is automated and controlled by MATLAB via graphical user interface (GUI). We provide a complete description of the setup in Methods.

### Optimizing the printing process

**Figure 2:**
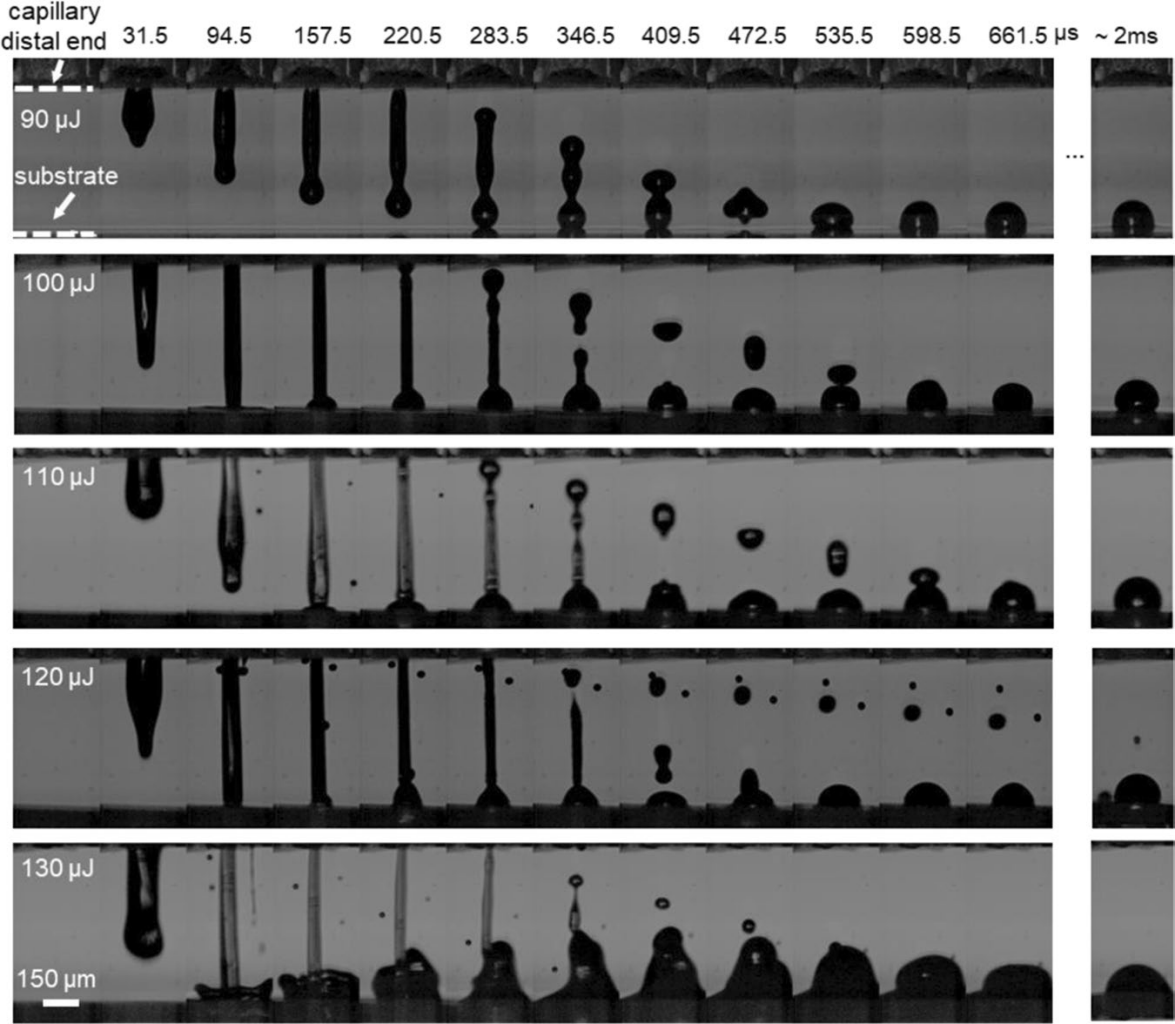
Sequences of snapshots showing micro-jet evolution and drop formation for different laser energies. The laser pulse was focused at the middle point of the capillary and 500 μm above its distal end.

**Figure 3:**
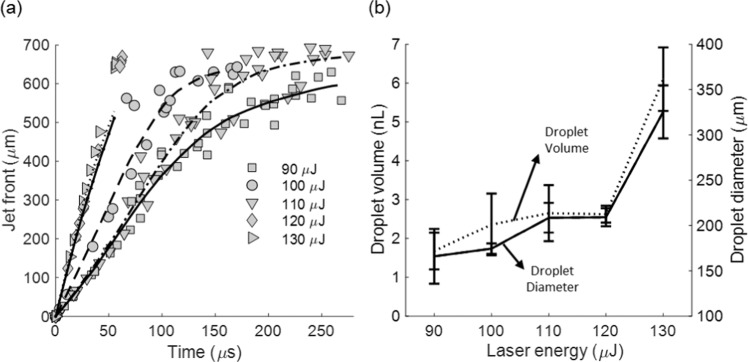
(**a**) The dependence of the bio-ink jet front position on the laser energy. Gray symbols represent data points and black lines represent the best fitted curve. N = 10 (per energy). (**b**) The dependence of the droplet volume (dotted line) and droplet diameter (solid line) on the laser energy. N = 10 (per energy).

In the first part of our work we sought to study and optimize the printing process via the visualization of the bio-ink ejection from the microcapillary tip. Our primary objective was to determine the laser energy resulting in the deposition of uniform cell-containing droplets on fibrin receiving substrates. We varied the laser energy from 90 μJ (i.e., ejection threshold) to 130 μJ and found that the bio-ink is ejected in the form of a micro-jet that eventually reaches the substrate (Fig. 2). The jetting behaviour is similar to that observed in previous studies on laser-induced flow focusing of model solutions for drug delivery applications^23,24^. However here, the bio-ink jets are less energetic and do not penetrate the substrate. Their impact to the substrate results in the formation of an oscillating droplet that can even bounce back for low energies (90 μJ). For higher energies (120 and 130 μJ), satellite droplet formation as well as “splashing” behavior can be observed. Note that the deposited droplets “relax” at different contact angle depending on the laser energy. Similar phenomena have been widely observed in the LIFT literature^25,26^. Figure 3a illustrates the jet-front position extracted from the jet-ejection visualization. The average jet-front velocity ranges from 3.2 to 11.60 m/s for the examined laser energies (90 to 130 µJ). The average jet-front velocity is constant with time for high energies. However, jet-front slowing with time is observed for low ejection velocities (90 to 110 µJ), indicating the predominant effect of viscous and surface forces on the ejection process. Similar behavior has been reported for LIFT generated micro-jets^27^. The diameter of the deposited droplets varied from 165 to 325 µm for the examined laser energy (Fig. 3b). The corresponding droplet volume varied from 1.675 to 6.1 nL (Fig. 3b). It was calculated using the contact angle of the deposited droplets at relaxation around 2 ms (see last column in Fig. 2).

**Figure 4:**
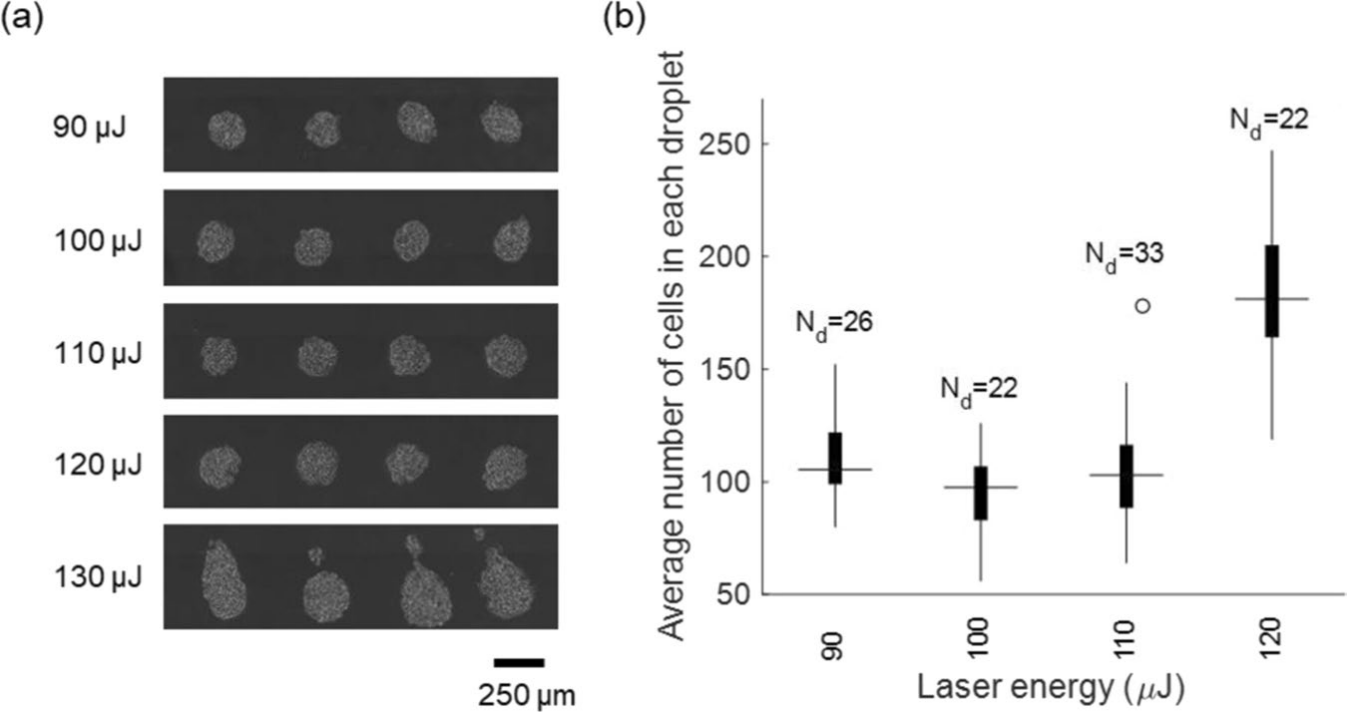
(**a**) Optical microscopy images of LIST-printed HUVECs for various laser enegies. (**b**) The number of the cells per droplet for various laser energies.

We acquired optical microscopy images of LIST-printed droplets 30 minutes post printing to evaluate the printing quality and to measure the number of HUVECs contained in each droplet (Fig. 4a). We found no significant change in the circularity of the deposited droplets within the 90 to 120 µJ laser printing energy range. However, we observed non-circular drops and satellite droplet deposition for 130 µJ (Fig. 4a). We found that the number of HUVECs per drop ranged from 105 ± 47 to 175 ± 66 for the examined laser energies (90 to 120 µJ) (Fig. 4b). These findings agree with fast imaging, where “splashing” behavior was observed for high laser energy printing (Fig. 2).

Compared to micro-jet generation by laser-induced flow focusing^20^, we used similar laser energy density threshold at focus to generate stable micro-jets. The generated cell-containing micro-jets present slightly lower threshold ejection speed (3.2 m/s) compared to that (4–7 m/s) observed for model solutions in ref. ^20^. Contrary to work exploiting flow focusing effects^20,23,24^, we do not apply hydrophobicity enhancement treatment to the microcapillary tip. Therefore, there is almost no meniscus concavity to provoke such effects. Nevertheless, uniform printing is demonstrated in absence of flow-focusing. Furthermore, the spatial resolution for our cell-bioprinting setting is 165 μm, similar to the one attained for model solution printing in ref. ^20^ but lower than the one (10–140 µm) attained by LIFT for similar cell types^28–31^. The use of microcapillaries of smaller size can potentially further improve the spatial resolution in LIST. Note that in this work we used a constant 500 μm distance between the laser focus position and the capillary distal end. Given that this distance has been shown to affect jetting dynamics in flow-focusing applications^21^, further optimization of LIST might be attained by fine tuning this parameter for cell bio-bioprinting.

### LIST printed HUVECs maintain their ability to migrate and proliferate

The preservation of the cell migration and proliferation characteristics is central for bio-printing applications. We used live-cell time lapse imaging to assess the behavior of LIST-printed HUVECs. We focused on laser printing at 100 μJ because it resulted in the deposition of uniform and reproducible droplets in the optimization study. HUVECs-containing drops were printed at a separation distance of 500 µm and followed for 3 days by optical microscopy. At day 3, we stained with Calcein AM and Hoechst 33342 to access cell viability. We found that LIST-printed HUVECs progressively migrated from the initial area of deposition towards distant areas of the fibrin gel (Supplementary Fig. S1a–c). At day 3, the cells reached high confluency and covered uniformly the surface of the fibrin gel. Fluorescence imaging at day 3 indicated high cell viability (98%) post printing (Supplementary Fig. S1d–f). These results indicate that LIST printed HUVECs maintain their ability to migrate and proliferate.

### LIST printed HUVECs present marginal loss of viability compared to control deposited HUVECs

**Figure 5:**
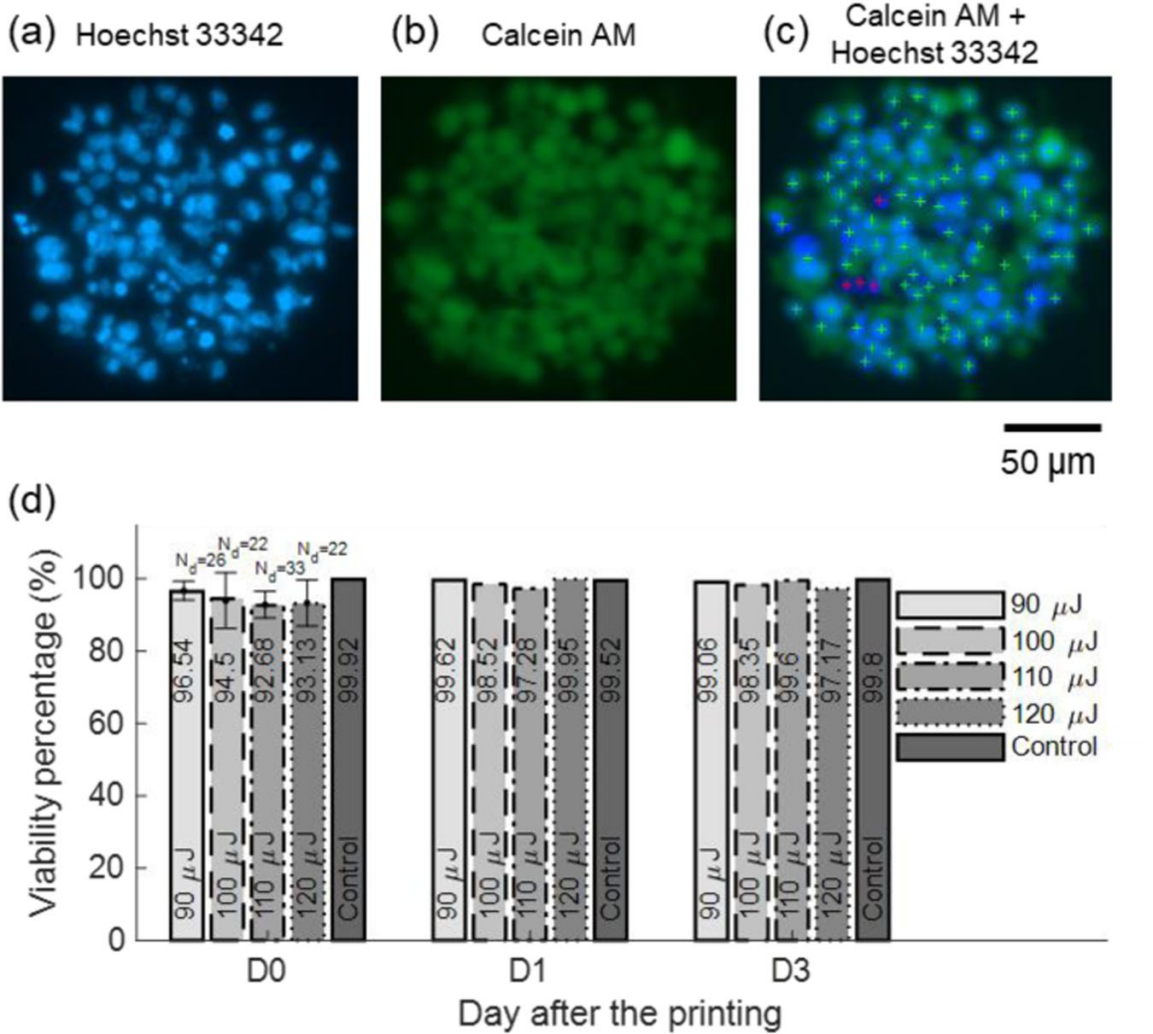
(**a,b**) Fluorescence microscopy images of LIST printed cells at 90 μJ. (**c**) Combined imaging channels, including algorithm-generated cell labeling marks. Green crosses indicate live cells and red crosses indicate dead cells. (**d**) The dependence of the HUVEC cell viability on the laser energy for 0, 1 and 3-days post printing. N_d_ indicates the number of droplets.

Given that LIST involves direct irradiation of a small section of the bio-ink, we sought to quantify potential effects on the viability of the deposited cells. We printed multiple droplets by varying the laser energy from 90 to 120 µJ. We used the viability assay described in Methods to measure cell viability at 0, 1, and 3 days post printing. Figures 5a–c show the typical steps implemented by the cell viability quantification algorithm. Hoechst 33342 stains the nucleus of all cells (Fig. 5a) and facilitates automated cell counting and cell coordinates registration (see crosses in Fig. 5c). The cell coordinates are used to interrogate the intensity of Calcein AM (staining live cells only) in the green channel. The positions of dead cells in the combined channels image are indicated by red crosses (Fig. 5c). Right after printing, we found that the cell viability varied from 96.5% to 93.1% for the examined laser range (90 to 120 μJ). There is marginal decrease in cell viability due to increase in laser energy. This can be explained by the increased thermomechanical impact on the cells at high energies compared to low energies, including higher pressure and temperature inside the capillary and generation of higher impact pressures upon jet collision to the fibrin gel. For days 1 and 3, cell viability increased up to the control level. This is explained by the fact of not considering the cell division rate in our quantification. These results indicate that LIST has only a marginal effect on the viability of the printed cells for the examined laser energy. Similar viability has been observed for printing of HUVECs by LIFT^28,29^ (i.e. >90%). LIST involves direct irradiation of a small fraction of the deposited cells. Further studies are required to evaluate potential mutagenic effects on those cells. Genotoxic effects have been observed *in-vitro* for laser irradiation of fibroblasts at 3 J/cm^2^ (532 nm) and at 10 J/cm^2^ (1064 nm)^32^. In this work we used 532 nm and exceeded this threshold at the focal point; thus, a tiny fraction of the deposited cells might be affected. Note that the 1064 nm wavelength presents not only higher threshold for the occurrence of genotoxic effects but also lower cavitation threshold in water compared to 532 nm. Future work on LIST at 1064 nm could eliminate the need to use a radiation absorber in the bio-ink and minimize potential mutagenic effects.

### LIST-printed HUVECs form intracellular junctions

**Figure 6:**
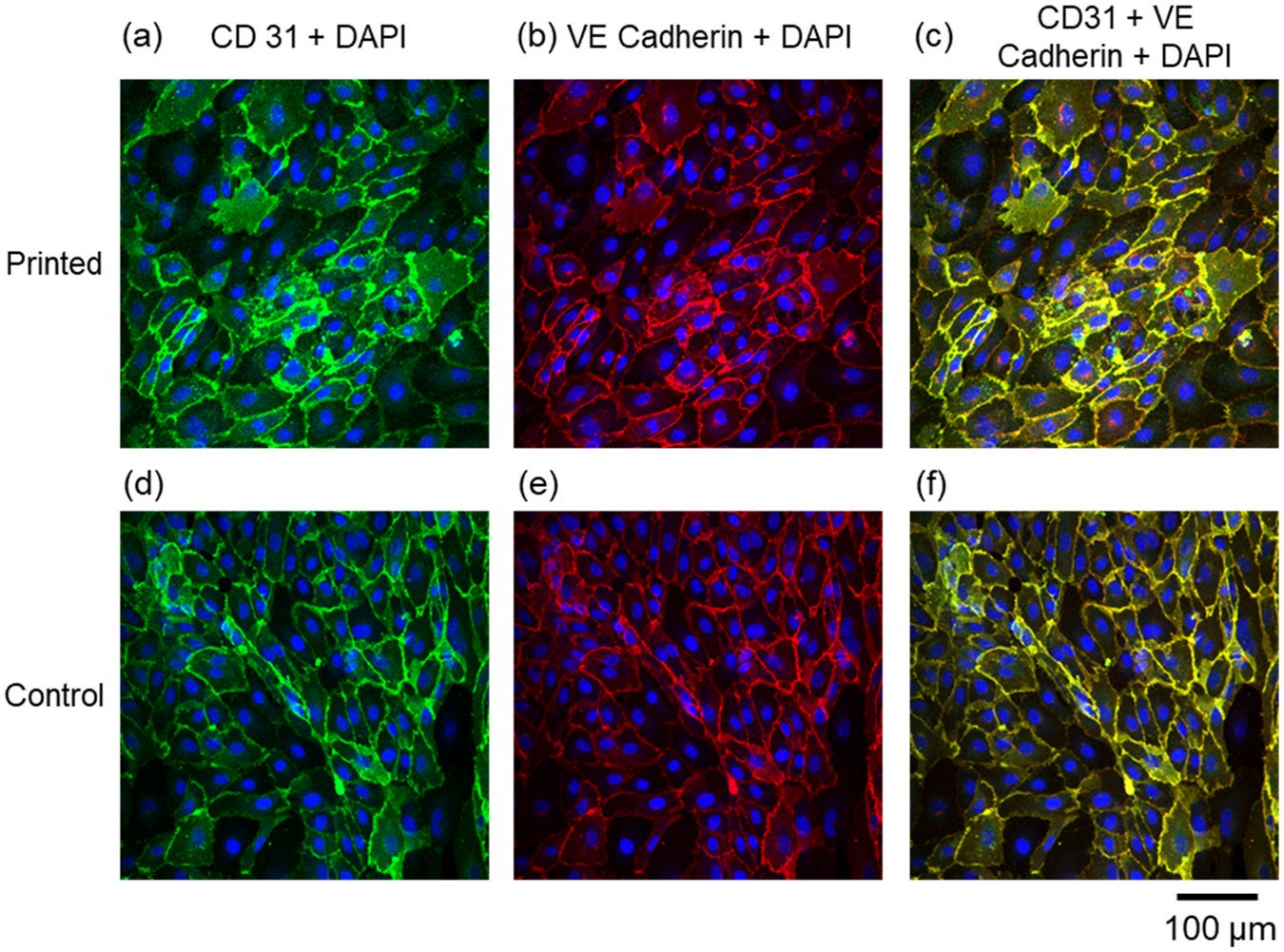
Confocal microscopy images of (**a**–**c**) LIST-printed (100 μJ) and (**d**–**f**) control HUVECs. Green indicates CD31 staining, red shows VE Cadherin and blue indicates cell nuclei staining with DAPI.

Cultured endothelial cells such as HUVECs are known to form intercellular junctions. These junctions are composed of several cell adhesion molecules including PECAM-1/CD31, a cell adhesion and signaling molecule, and VE-cadherin, which has is essential for the formation of endothelial adherens junctions. We sought to investigate whether proper intracellular junctions were formed between LIST-printed HUVECs. We LIST-printed HUVECs at 100 μJ. 3-days post printing, the cells formed a relatively uniform and confluent layer on the fibrin gel. We performed immunofluorescence imaging to interrogate the presence of intercellular junctions (VE-cadherin and CD31) in both LIST-printed and control HUVECs (Fig. 6). We found that LIST-printed HUVECs form intercellular junctions similar to control HUVECs cells. In fact, there was no apparent difference in the intensity and/or spatial distribution of the junction observed for the two groups. These results indicate the LIST-printed cells preserve their angiogenic junctional phenotype.

### High speed LIST printing

**Figure 7:**
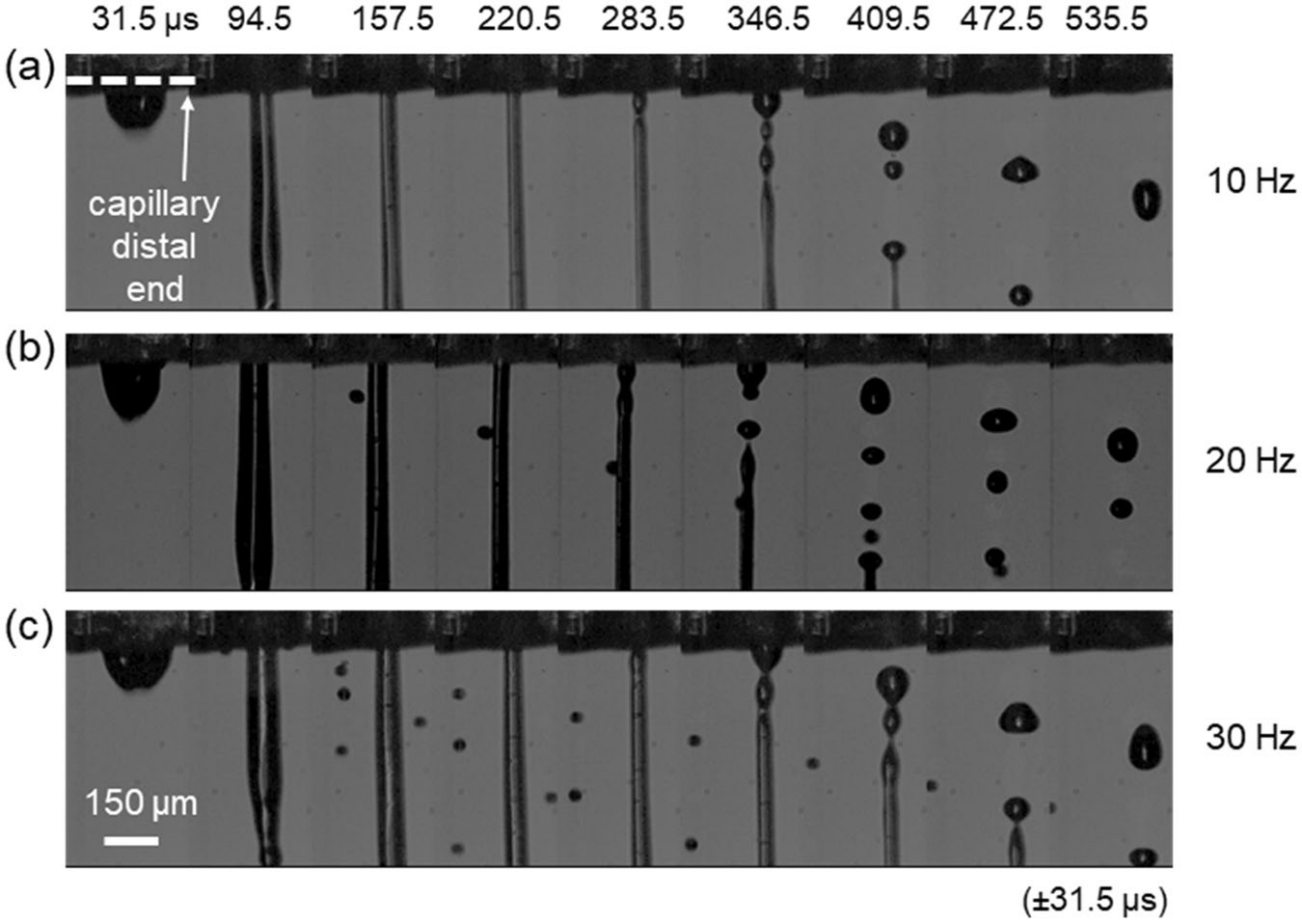
Sequences of snapshots showing micro-jet evolution for (**a**)10 Hz (**b**) 20 Hz and (**c**) 30 Hz. The laser energy was kept constant at 100 µJ. The laser pulse was focused at the middle point of the capillary and 500 μm above its distal end.

**Figure 8:**
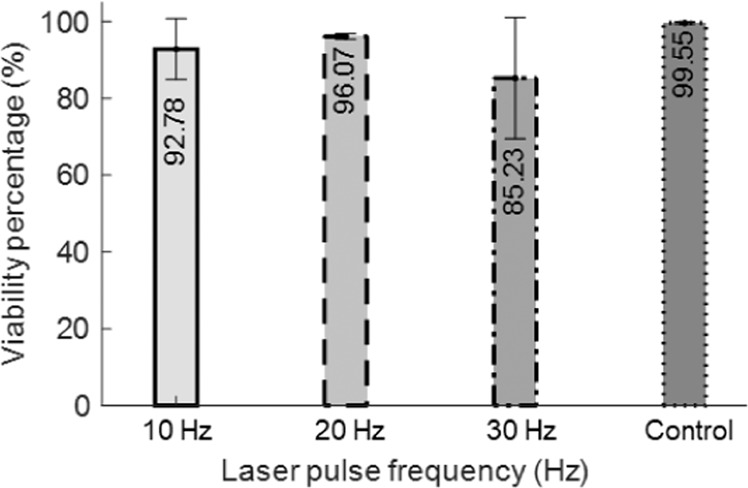
The dependence of the HUVEC cell viability on the printing speed. The laser energy was kept constant at 100 µJ.

Efficient printing of clinically relevant constructs (i.e., size > 1 cm^3^) in a reasonable time period requires high-speed printing. In this context, we sought to study printing speed capabilities in LIST. We examined how the increase in the printing speed affects the jetting dynamics and the viability of the deposited cells. We increased the printing speed up to 30 Hz, which was the maximum repetition rate of our laser. We kept the laser energy constant (100 μJ) for this series of experiments and we did not use any substrate to prevent the perturbation of the ejected jets by already deposited material. The ejected jets showed similar spatiotemporal evolution for the tested printing speeds of 10, 20 and 30 Hz (Fig. 7). However, for 30 Hz we observed the ejection of small satellite droplets around the main jet. We found insignificant differences on the jet-front ejection speed, i.e., 5.2 m/s for 1 Hz, 4.2 m/s for 10 Hz, 5.5 m/s for 20 Hz and 5.0 m/s for 30 Hz. Moreover, we found that the microjet detachment occurs at a relatively constant time point for the tested conditions i.e., from 315 to 378 μs. This indicates a potential printing speed up to 2.5 kHz. Indicatively, for LIST-printing at 100 μJ, one would need ~236 min to print a 1 cm^3^ construct at 30 Hz and 2.83 min to print the same at 2.5 kHz. We further examined whether the increase of the printing speed affects the viability of the HUVECs. We found that the differences in the cell viability for 10, 20 and 30 Hz lied within the experimental error (Fig. 8). These results indicate that with appropriate technical modifications, LIST has the potential to reach high printing speeds up to the range achieved by ink-jet printing.

## Conclusions

We developed and validated a laser-assisted drop-on-demand method to print cell-containing droplets. Under optimal printing conditions (laser energy: 100 μJ), uniform HUVECs containing droplets were deposited on fibrin coated substrates. Post printing, HUVECs maintain high viability and preserve their angiogenic junctional phenotype. The minimum droplet size was 165 μm for the tested conditions. Printing of smaller droplets should be possible by using thinner microcapillaries and/or by tuning the viscosity of the bio-ink. Here we validated LIST using a low-viscosity fibrinogen-based cell bio-ink. However, LIST can be adapted for printing of medium and high-viscosity bio-inks. The printability range as well as the effect of the viscosity on the printing outcome (resolution, cell viability, maximum repetition rate) are yet to be determined. We showed droplet bio-printing up to a 30 Hz repetition rate, i.e., equal to the maximum repetition rate of the available laser. However, fast imaging of jet ejection dynamics indicate that LIST can potentially reach a printing speed of 2.5 kHz. Similar to ink-jet printing, simultaneous printing of multiple bio-inks is technically possible using multiple microcapillaries. LIST is technically uncomplicated and can foster 3D printing applications. It can potentially cover a technological gap in bioprinting technologies, between ink-jet printing and LIFT, as it could not only print bio-inks of high-viscosity but also support 3D printing of constructs with clinically relevant size.

## Methods

### Laser beam delivery system

Figure 1b shows a schematic of the beam delivery system. We first expanded the exiting laser (Nano L series, Litron Lasers) beam from 4 mm to 8 mm in diameter by using a pair of concave (f = −50 mm, LC1715-A-ML, Thorlabs) and convex lenses (f = 100 mm, LA1509-A, Thorlabs). We used a motorized optical attenuator to control the laser energy, composed of a half-wave plate (WPMH05M-633, Thorlabs), a rotation stage (PRM1Z8, Thorlabs) and a polarizing beam splitter. On-line laser energy monitoring was attained by sampling the beam with a beam splitter (10:90 (R:T), BSN10, Thorlabs) and by measuring the energy of the sampled beam with a pyroelectric sensor (QE12LP-S-MB, Gentec-eo) to derive the energy at the sample. A second pair of lenses (f = −50 mm, LC1715-A-ML, and f = 150 mm, LA1433-A-ML, Thorlabs) was used to further expand the beam from 8 mm to 24 mm in dimeter and a pair of broadband dielectric mirrors (BB1-E02, Thorlabs) to elevate it to the desired level. Finally, the beam was focused at the middle of the capillary (Vitrocom hollow square capillary, inner size 0.3 mm × 0.3 mm, 0.15 mm wall thickness, 50-mm long) by using a 4X objective lens (plan achromat, NA = 0.1, Olympus).

### Drop-on-demand unit

The drop-on-demand unit uses a mechanical shutter (SH05, Thorlab) and an xyz motorized translational stage (PT1-Z8 + MAX 201, Thorlabs) to control the ejection of the droplets and their positioning on the receiving substrate (Fig. 1b). The capillary is fixed during printing and the receiving substrate is displaced according to the desired printing pattern. A syringe pump (NE-1000, New Era Pump Systems Inc.) was used to refill the capillary at regular intervals.

### Micro-jet visualization system and analysis

We used shadowgraphic imaging to study the micro-jet ejection dynamics in LIST (Fig. 1b). Two approaches were implemented: (i) long-exposure imaging and (ii) fast-imaging. The first approach enabled the acquisition of single blur snapshots of microjets at pre-determined time points regarding laser firing (accuracy: ±1 μs), while the second approach enabled the acquisition of multiple jet snapshots of microjet evolution at loosely determined time points (accuracy: ±31.5 μs). By combining the two approaches we reconstructed the complete jet ejection dynamics for given laser conditions. For both approaches, we used a high-speed camera (Chronos 1.4, Kron Technologies), an achromatic lens (AC254-150-A-ML, Thorlabs)  and back LED illumination (MCWHL5, Thorlabs). A delay generator (DG535, Stanford Research Systems) and a photodiode (DET10A, Thorlabs) were used to synchronize the laser with the camera at desired time delays. For long-exposure imaging, we set the exposure time to 50 µs. For high-speed imaging, we set the frame rate to 15870 fps (period 63 µs) and the exposure time to 3µs. For this imaging mode, the first frame had an arbitrary delay (0 to 63 µs) with respect to laser firing. We first used fast imaging to acquire multiple snapshots of jet dynamics generated at different energies. Then, we used long-exposure imaging at the same energies to estimate the speed of the ejected jets (Supplementary Fig. S2). By knowing the jet ejection speed for a given energy, we assigned an approximate time stamp to the first frame of fast imaging (Supplementary Fig. S2). Finally, we used MATLAB to process time-resolved images to extract the jet front as a function of time.

### Bio-ink formulation

HUVECs (Promocell) were cultured in EndoGRO-VEGF medium (Millipore). For the bio-ink we used 10^6^ HUVECs per ml suspended in Basal medium (EndoGRO, Millipore), supplemented with fibrinogen (13.24 µM) (F8630-5G; Sigma-Aldrich) and aprotinin (7.68 µM) (10820–25MG; Sigma-Aldrich) that facilitated the gelation processes post printing. A red food dye, Allura red AC (458848-100G, Sigma-Aldrich), was also added to a final concentration of 10 mΜ to enhance light absorption by the bio-ink.

### Printing substrates

We used fibrin-coated 24 mm × 50 mm microscope cover glasses (12-545-F, Fisher Scientific) as printing substrates. For the fibrin gel coating (~1 mm-thick), we used 1185 µL of a Basal medium (SCME001, Millipore), containing fibrinogen (13.24 µM) (F8630-1G, Sigma) an aprotinin (7.68 µM) (10820-25MG; Sigma-Aldrich) and 15 µL of a thrombin solution (1.25 U/mL final concentration in the fibrin gel) (T7513-100UN, Sigma-Aldrich). We used drop-casting to deposit the two solutions onto the microscope cover glasses one hour before printing.

### Printing protocol

Freshly prepared bio-ink (~100 μL) was loaded to the squared capillary using the syringe pump. The laser beam was focused in the middle of the capillary, 500 µm far from its distal end. The receiving substrate was fixed on an xyz translation stage and placed 500–700 µm far from the capillary tip. Laser energies at the sample varied from 90 to 130 μJ. Printing patterns consisted in arrays of individual droplets separated by a 500 μm gap. After printing, samples were placed in an incubator (37 °C, 5% CO_2_) for 5–10 minutes. Next, we rinsed the samples twice with EndoGRO-VEGF medium (Millipore) to dilute the light absorbing red dye and put them back in the incubator till further analysis.

### Viability assay

We used a Calcein AM viability assay to access the viability of the printed cells at different time points and for various printing conditions. Post printing, we used Hoechst 33342 (14.237 µM) (14533–100MG; Sigma-Aldrich) to stain all printed cells and Calcein AM (0.402 µM) (400146, Cayman chemical) to evaluate the presence of live cells. Fluorescence images were acquired by an inverted motorized microscope with live cell imaging capabilities (Zeiss AxioObserver Z1). We developed a MATLAB algorithm to process the images. The algorithm detects the nucleus of all printed cells, stained in blue by Hoechst 33342. For each cell, the intensity I_c_ at the green channel (Calcein AM) is registered. The background green channel intensity, I_b_, as well as its standard deviation, σ_Ib_, are considered. A cell is considered live (i.e., Calcein AM positive) if its green channel intensity satisfies the following formula I_c_ > I_b_ + 5 × σ_Ib_.

### Visualization of intercellular junctions

We used immunofluorescence to visualize intercellular junctions for LIST-printed and control deposited HUVECs 3-days post printing/deposition. We first incubated the samples with PFA 4% for 10 to 15 minutes to fix the cellular protein and subcellular structures in place. The samples were then incubated with a blocking solution containing 3% BSA and 0.1% Triton X-100 in PBS (including Mg2+ and Ca2+) for 10–15 minutes to induce permeabilization. Samples were then incubated with CD 31 (1:500) and VE-Cadherin (1:40) antibodies diluted in permeabilization medium at 4 °C in the dark overnight. The following day, the samples were treated with the secondary antibody (Alexa Fluor 647 chicken anti-rat) (1:200) for 3 hours in room temperature. Finally, the samples were imaged by an upright confocal microscope (Zeiss AxioExaminer Z1).

## Supplementary information

Supplementary Information.

## References

[1] Ozbolat, I. T. *3D Bioprinting: Fundamentals, Principles and Applications*. *3D Bioprinting: Fundamentals, Principles and Applications* (2016).

[2] Murphy SV, Atala A (2014). 3D bioprinting of tissues and organs. Nat. Biotechnol..

[3] Gudapati H, Dey M, Ozbolat I (2016). A comprehensive review on droplet-based bioprinting: Past, present and future. Biomaterials.

[4] Xu T, Jin J, Gregory C, Hickman JJ, Boland T (2005). Inkjet printing of viable mammalian cells. Biomaterials.

[5] XU T (2006). Viability and electrophysiology of neural cell structures generated by the inkjet printing method. Biomaterials.

[6] Antoshin AA (2019). LIFT-bioprinting, is it worth it?. Bioprinting.

[7] Panwar, A. & Tan, L. P. Current status of bioinks for micro-extrusion-based 3D bioprinting. *Molecules* **21** (2016). 10.3390/molecules21060685 PMC6273655 27231892

[8] Schiele NR (2010). Laser-based direct-write techniques for cell printing. Biofabrication.

[9] Boutopoulos C, Andreakou P, Kafetzopoulos D, Chatzandroulis S, Zergioti I (2008). Direct laser printing of biotin microarrays on low temperature oxide on Si substrates. Phys. Status Solidi.

[10] Boutopoulos C, Touloupakis E, Pezzotti I, Giardi MT, Zergioti I (2011). Direct laser immobilization of photosynthetic material on screen printed electrodes for amperometric biosensor. Appl. Phys. Lett..

[11] Colina M, Serra P, Fernández-Pradas JM, Sevilla L, Morenza JL (2005). DNA deposition through laser induced forward transfer. Biosens. Bioelectron..

[12] Catros S (2012). Layer-by-Layer Tissue Microfabrication Supports Cell Proliferation *In Vitro* and *In Vivo*. Tissue Eng. Part C Methods.

[13] Koch L (2012). Skin tissue generation by laser cell printing. Biotechnol. Bioeng..

[14] Kattamis NT, Purnick PE, Weiss R, Arnold CB (2007). Thick film laser induced forward transfer for deposition of thermally and mechanically sensitive materials. Appl. Phys. Lett..

[15] Barron JA, Krizman DB, Ringeisen BR (2005). Laser printing of single cells: Statistical analysis, cell viability, and stress. Ann. Biomed. Eng..

[16] Lin Y, Huang G, Huang Y, Tzeng T-RJ, Chrisey D (2010). Effect of laser fluence in laser-assisted direct writing of human colon cancer cell. Rapid Prototyp. J..

[17] Guillemot F, Souquet A, Catros S, Guillotin B (2010). Laser-assisted cell printing: Principle, physical parameters versus cell fate and perspectives in tissue engineering. Nanomedicine.

[18] Koch L, Gruene M, Unger C, Chichkov B (2013). Laser Assisted Cell Printing. Curr. Pharm. Biotechnol..

[19] Koch L (2010). Laser printing of skin cells and human stem cells. Tissue Eng. - Part C Methods.

[20] Delrot P, Modestino MA, Gallaire F, Psaltis D, Moser C (2016). Inkjet Printing of Viscous Monodisperse Microdroplets by Laser-Induced Flow Focusing. Phys. Rev. Appl..

[21] Tagawa Y (2012). Highly focused supersonic microjets. Phys. Rev. X.

[22] Peters IR (2013). Highly focused supersonic microjets: numerical simulations. J. Fluid Mech..

[23] Tagawa Y, Oudalov N, El Ghalbzouri A, Sun C, Lohse D (2013). Needle-free injection into skin and soft matter with highly focused microjets. Lab Chip.

[24] Kiyama A (2019). Visualization of penetration of a high-speed focused microjet into gel and animal skin. J. Vis..

[25] Boutopoulos C, Papageorgiou DP, Zergioti I, Papathanasiou AG (2013). Sticking of droplets on slippery superhydrophobic surfaces by laser induced forward transfer. Appl. Phys. Lett..

[26] Boutopoulos C, Chatzipetrou M, Papathanasiou AG, Zergioti I (2014). Time-resolved imaging and immobilization study of biomaterials on hydrophobic and superhydrophobic surfaces by means of laser-induced forward transfer. Laser Phys. Lett..

[27] Brown MS, Brasz CF, Ventikos Y, Arnold CB (2012). Impulsively actuated jets from thin liquid films for high-resolution printing applications. J. Fluid Mech..

[28] Kawecki, F. *et al*. Self-assembled human osseous cell sheets as living biopapers for the laser-assisted bioprinting of human endothelial cells. *Biofabrication* **10** (2018). 10.1088/1758-5090/aabd5b 29638221

[29] Pirlo RK, Wu P, Liu J, Ringeisen B (2012). PLGA/hydrogel biopapers as a stackable substrate for printing HUVEC networks via BioLP^TM^. Biotechnol. Bioeng..

[30] Ovsianikov, A. *et al*. Laser printing of cells into 3D scaffolds. *Biofabrication* **2** (2010). 10.1088/1758-5082/2/1/014104 20811119

[31] Guillotin B (2010). Laser assisted bioprinting of engineered tissue with high cell density and microscale organization. Biomaterials.

[32] Senturk N (2010). Genotoxic effects of 1064-nm Nd:YAG and 532-nm KTP lasers on fibroblast cell cultures: Experimental dermatology. Clin. Exp. Dermatol..

