# Supplementary material for: Drop-on-demand cell bioprinting via Laser Induced Side Transfer (LIST)
Source: Sci Rep. 2020 Jun 16;10:9730. doi: 10.1038/s41598-020-66565-x (PMC7298022; doi:10.1038/s41598-020-66565-x)
Supplement: Supplementary file 1 — Supplementary Information. [file 41598_2020_66565_MOESM1_ESM.pdf]

Supplementary Material

Drop-on-demand cell bioprinting via Laser Induced Side Transfer (LIST)

Hamid Ebrahimi Orimi[1,2], Sara Hosseini[1,6], Erika Hooker[1,3,5], Sivakumar Narayanswamy[2,4], Bruno Larrivee[1,3,5], Christos Boutopoulos[1,3,6*]

[1]Centre de Recherche Hôpital Maisonneuve-Rosemont, Montréal, Canada

[2]Department of Mechanical, Industrial and Aerospace Engineering, Concordia University, Montréal, Canada

[3]Department of Ophthalmology, Faculty of Medicine, University of Montreal, Montréal, Canada

[4]Departent of Mechanical Engineering, SRM University, AP, Amaravati, India

[5]Department of Molecular Biology, University of Montreal, Montreal, Quebec, Canada.

[6]Institute of Biomedical Engineering, University of Montreal, Montreal, Quebec, Canada

*christos.boutopoulos@umontreal.ca

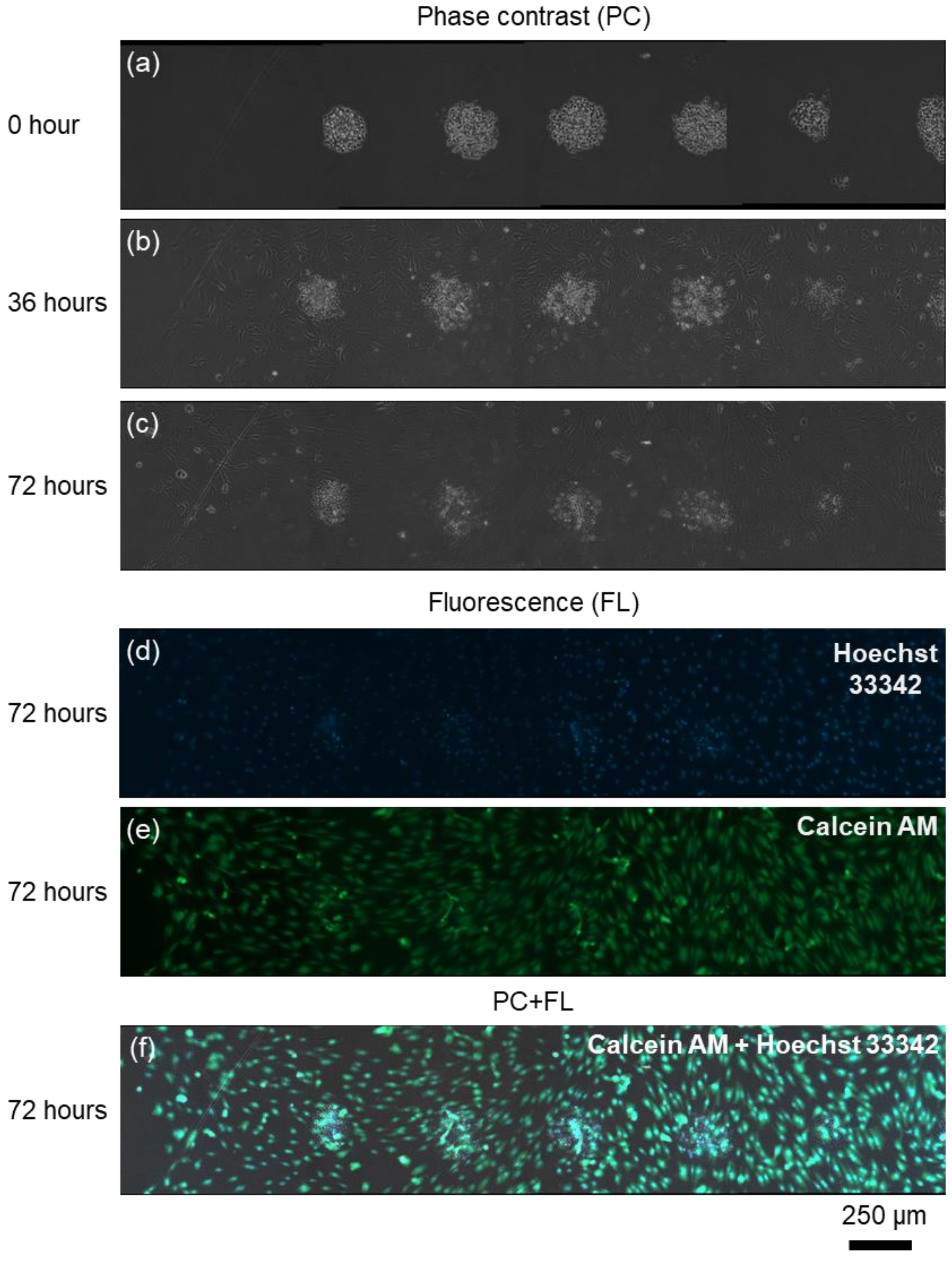

Figure S1. HUVECs migration post LIST printing. (a-c) Phase contrast (PC) optical microscopy images of LIST-printed (100 μJ) HUVECs 0 h, 36 h and 72 h post printing. (d-e) Fluorescence (FL) microscopy and (f) combined BF/PC images 72 h post printing. Calcein AM (green) stains live cells and Hoechst 33342 (blue) stains all cells.

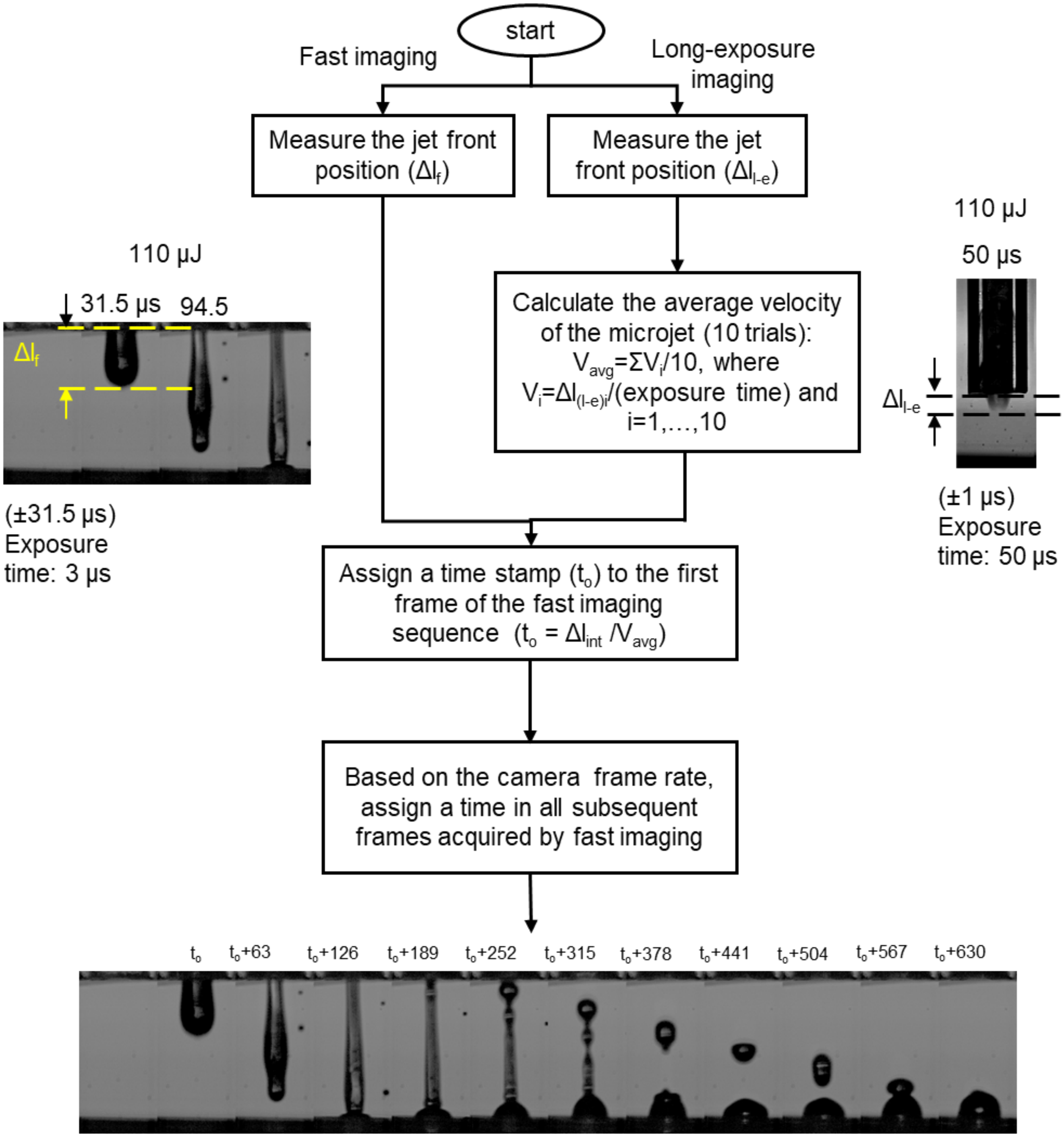

Figure S2. Flow chart showing the steps implemented to assign a time point to the first frame of an image sequence acquired by fast imaging.
